# Avoiding soy foods and certain nuts and seed foods in the management of severe cyclical mastalgia: a case report

**DOI:** 10.3389/fgwh.2025.1655250

**Published:** 2025-12-18

**Authors:** Kaifeng Deng, Qiaoyun Zhang, Ruzhen Wei

**Affiliations:** Medical Science Laboratory, The Fourth Affiliated Hospital of Guangxi Medical University, Liuzhou, China

**Keywords:** soy, nut, seed, cyclical mastalgia, case report, hormone analogs

## Abstract

Severe cyclical mastalgia is a common condition that can substantially impair women's daily activities and quality of life. Pharmacological treatments are often used, but non-pharmacological strategies remain underexplored. We report a case of a 28-year-old woman with long-standing severe cyclical mastalgia and hyperprolactinemia, who achieved symptom resolution through strict avoidance of soy foods and certain nuts, while maintaining her usual lifestyle and diet otherwise. Hormonal measurements indicated decreases in premenstrual prolactin, progesterone and estradiol levels, and even minimal intake of these foods provoked mild breast discomfort. This case suggests a potential link between soy consumption and cyclical mastalgia in susceptible individuals and highlights the possibility of non-pharmacological dietary management. Further research is needed to assess the efficacy, safety, and broader applicability of this approach.

## Introduction

Cyclical mastalgia, generally considered a benign and self-limiting condition associated with the menstrual cycle, can nonetheless cause substantial physical and psychological distress in affected women. Moderate to severe cases frequently impair daily functioning and reduce quality of life. Clinical observations have consistently highlighted the role of hormonal regulation—particularly prolactin, estradiol, and progesterone in the onset and severity of symptoms (1, 2). The efficacy of certain drugs is closely associated with prolactin and estrogen. Danazole exhibits weak androgenic activity and inhibits ovarian steroid production, which reduces estrogen levels in the body and rapidly alleviates mastalgia symptoms. It has an effective rate of 79% for cyclical mastalgia and is suitable for patients with severe mastalgia. Bromocriptine, a dopamine agonist, effectively inhibits the synthesis and secretion of prolactin from the hypothalamus, with an effective rate of 54% for cyclical mastalgia (3). Tamoxifen is a steroidal anti-estrogen drug that is effective in 72% of patients with severe cyclical mastalgia (4). However, these medications can produce adverse effects, including menstrual irregularities and potential impacts on ovarian function. Non-steroidal anti-inflammatory drugs (NSAIDs) are frequently utilized as first-line agents for acute pain episodes due to their rapid onset and favorable safety profile. Additionally, phytotherapeutic agents and traditional Chinese medicines have gained increasing attention due to their relatively lower risk of adverse effects. Despite these interventions, it is difficult to achieve a radical cure for cyclical mastalgia. Given the complex pathophysiological mechanisms underlying cyclical mastalgia, investigators continue to pursue efficacious and well-tolerated therapeutic strategies.

Multiple studies have investigated soy foods’ influence on breast health. Studies have suggested that phytoestrogens exert a dual regulatory effect as weak estrogens and anti-estrogens in the body. When endogenous estrogen levels are low, phytoestrogens can bind to estrogen receptors to exert estrogen-like effects; when endogenous estrogen levels are high, they can competitively inhibit estrogen receptors on target cells to produce anti-estrogenic effects (5). Furthermore, studies have reported that soy foods, rich in phytoestrogens, may reduce the risk of breast cancer (6–8). Therefore, some women aim to improve breast health, regulate hormone levels in the body, and alleviate cyclical mastalgia by consuming legume-containing foods. In a study by Touillaud M et al., the consumption of soy supplements may reduce the risk of estrogen receptor-positive breast cancer but potentially increase the risk of estrogen receptor-negative breast cancer (9). This result indicates that the effects of phytoestrogens on the human body vary among individuals and warrant further in-depth investigation. More research may be needed to clarify the effects of legume consumption on breast health.

Given the intimate connection between endocrine function and breast tissue, it is plausible that dietary phytoestrogens may modulate cyclical mastalgia in susceptible individuals. The uterus and breast function as target organs of the endocrine system, whereby cyclical mastalgia often co-occurs with abdominal distension or dysmenorrhea. Herein, we report a distinctive case in which a patient successfully eliminated severe cyclical mastalgia and associated lower abdominal discomfort by strictly avoiding soy and certain nut products. This case underscores the potential for dietary modulation to influence hormonal profiles and symptom severity, highlighting the need for individualized management strategies in women with hormone-sensitive mastalgia.

## Case presentation

The patient was a 28-year-old unmarried woman with a body mass inde(BMI) of 19, presenting with a history of hyperprolactinemia, breast hyperplasia and cysts. Over the past decade, she suffered from severe cyclical mastalgia lasting 5–7 days, and accompanied by hyperprolactinemia and dysmenorrhea. She reported no history of smoking, alcohol consumption, or intake of honey, coffee, cold beverages, strong tea, or dietary supplements.

In November 2015, the patient sought medical attention after experiencing an extension of cyclical mastalgia to 14 days, twice the usual duration. Serological examination revealed a prolactin level of 2,197 mIU/L and an estradiol level of 2,472 pmol/L, while other serological test results, including liver function, kidney function, and tumor markers, were within normal ranges. The ultrasound examination reveals breast hyperplasia and cysts, with no malignant lesions detected. MRI of the pituitary gland ruled out adenoma. Following the medical consultation, the patient declined drug intervention.

**Figure 1:**
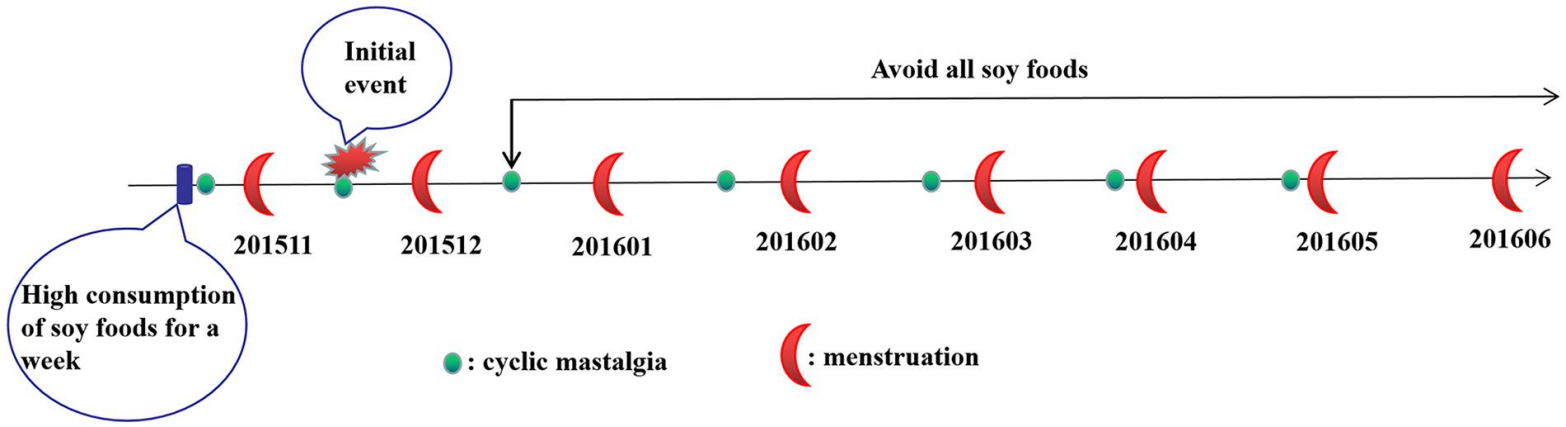
The timeline for symptom alleviation in cyclical mastalgia.

In December 2015, she experienced an earlier recurrence of cyclical mastalgia. As a medical worker, the patient recalled a unique event that she had congee mixed with various beans for breakfast and dinner continuously for a week in October 2015. She had never consumed a large amount of soy foods in a short time in the past. As soy foods are believed to help regulate women's endocrine system, she speculated that this dietary change might have exacerbated her symptoms. Consequently, she eliminated all soy products from her diet while maintaining her usual lifestyle, exercise, and daily schedule. After four months, cyclical mastalgia and dysmenorrhea were markedly alleviated. The timeline for symptom alleviation in cyclical mastalgia was shown in Figure 1. During the period of avoiding all soy foods, a small amount of seed foods(e,g soy sauce, pistachio, pumpkin seeds, peanut) could cause slight pain. Therefore, special attention should be paid to the food ingredient list.

So far, the patient had not experienced cyclic mastalgia again, and her menstrual cycle and physical condition are normal. Hormone levels were measured using Roche chemiluminescent reagents 3–5 days before menstruation. The values of serum prolactin, progesterone and estradiol had decreased to lower level in pre-menstrual period (Table 1). Observation indicated that excessive soy food consumption would increase the serum levels of prolactin, progesterone and estrogen and prolong the duration of cyclical mastalgia.

**Table 1:** The serum levels of prolactin, progesterone and estradiol during the premenstrual from 2014 to 2025.

Month/Year	02/2014	11/2015	03/2016	10/2016	2017	2018	03/2019	2020	2021	2022	03/2023	2024	04/2025
Prolactin (mIU/L)	2,088	2,197	978	1,049	N*	N	602	N	N	N	489	N	523
Progesterone (nmol/L)	76.81	87.33	50.18	40.77	N	N	38.62	N	N	N	42.56	N	45.50
Estradiol (pmol/L)	1,614	2,472	1,662	547	N	N	674	N	N	N	606	N	598

N*: No tested and no result. Prolactin: for non-pregnant women: 102–494; Progesterone: Follicular phase: 0.64–4.77, Ovulatory phase: 2.54–9.54, Luteal phase: 5.41–85.86. Estradiol: Follicular phase: 114–322, Ovulation phase: 222–1959, Luteal phase: 222–854.

Therefore, we proposed a hypothesis: does soy foods contain substances similar to prolactin, progesterone and estrogen? To explore potential mechanisms, pure seeds were ground into powder and dissolved in distilled water (without other matrices). After high-speed centrifugation, the supernatant was collected, and then Roche diagnostic reagents-originally used for detecting hormones in human serum were employed to detect compounds with hormone-like groups in the soy, nut and seed foods (Table 2). We detected estrogen analogues and progesterone analogues, but no prolactin analogue in these foods(Substances with chemical groups similar to those of hormones detected in legumes are referred to as hormone analogues). Due to limitations of the experimental conditions, other bioactive important substances in soy, nut and seed foods may also be involved in the effect of the increase of hormone level, which requires further in-depth research.

**Table 2:** The detection of prolactin analogues, progesterone analogues and estradiol analogues in these seeds.

Tested foods	Soy bean	Mung bean	Kidney bean	Pistachio	Pumpkin seeds	Peanut	Red bean	Yellow corn
Prolactin analogues^a^	0	0	0	0	0	0	0	0
Progesterone analogues^a^	**+**	**+**	**+**	**+**	**+**	**+**	**+**	**+**
Estradiol analogues^a^	**+**	**+**	**+**	**+**	**+**	**+**	**+**	**+**

a Prolactin analogues, progesterone analogues and Estradiol analogues: they were detected by the reagents of prolactin, progesterone and estradiol. Prolactin detection limit: 2 μmol/L; Progesterone detection limit: 0.159 nmol/L; Estradiol detection limit: 18.4 pmol/L. If the value exceeds the detection limit, it is marked as “+”.

Additionally, after the cyclic mastalgia subsided, prolactin required a longer time than estradiol to return to the lower level. This suggests that the increase of prolactin was likely a secondary response to dietary stimulation rather than a primary driver of breast pain. Moreover, minimal intake of seed foods could induce mild premenstrual breast discomfort. This case indicates a causal relationship between soy foods and the patient's cyclical mastalgia.

## Discussion and conclusion

This case demonstrates that complete avoidance of soy and certain other seed foods successfully eliminated cyclical mastalgia in a patient with a decade-long history of severe symptoms. These observations suggest that soy consumption may induce breast pain and alter hormonal profiles in susceptible individuals, highlighting that some patients may not derive universal benefits from soy and seed foods.

Traditionally, most patients with elevated prolactin were managed with medications; this case provides preliminary evidence that a non-pharmacological dietary intervention, specifically strict soy avoidance, may offer an alternative approach under appropriate clinical supervision. The marked improvement in breast pain and dysmenorrhea symptoms indicates that soy components influence both mammary and uterine tissues. The hormone-elevating effect induced by legume consumption in the human body may have implications for the management of gynecological diseases, as prior research has demonstrated that soy can modulate gene expression in breast cancer (10). Individual responses to seed foods may vary due to genetic or physiological factors. Among the female population, most individuals can likely consume and metabolize soy foods well. However, a small subset of individuals, such as some women with cyclical mastalgia, may be unable to tolerate soy foods due to individual genotypes or current physiological factors. Therefore, we aim to help this small group of women who are intolerant to soy foods achieve a comfortable physical state.

Clinical trials for this intervention have not yet been conducted due to the consideration regarding fertility and safety. For the general population, seed foods including soy and nut foods are prevalent in people's daily diets and avoidance requires substantial dietary vigilance. The patient also noted that avoiding soy foods requires significant self-discipline, given its widespread use as an ingredient in various foods and condiments. As this intervention was self-initiated, annual hormone level monitoring was not conducted after the resolution of cyclical mastalgia.

Given the multifactorial etiology of cyclical mastalgia, this approach may not be suitable and effective for all patients. Additionally, the long-term physiological effects of strict soy avoidance remain uncertain. Therefore, further research is required to validate the safety, efficacy, and generalizability of this non-pharmacological strategy in broader patient populations.

## Data Availability

The datasets presented in this study can be found in online repositories. The names of the repository/repositories and accession number(s) can be found in the article/Supplementary Material.
